# A randomized controlled trial evaluating the effect of low-dose chlormadinone in patients with low-risk prostate cancer: PROSAS study

**DOI:** 10.1093/jjco/hyab162

**Published:** 2021-10-26

**Authors:** Mikio Sugimoto, Yoshiyuki Kakehi, Shigeo Horie, Yoshihiko Hirao, Hideyuki Akaza

**Affiliations:** Department of Urology, Kagawa University Faculty of Medicine, Kagawa, Japan; Kagawa University, Kagawa, Japan; Department of Urology, Juntendo University Faculty of Medicin, Tokyo, Japan; Osaka Gyoumeikan Hospital, Osaka, Japan; Interfaculty Initiative in Information Studies, Graduate School of Interdisciplinary Information Studies, The University of Tokyo, Tokyo, Japan

**Keywords:** active surveillance, chlormadinone acetate, persistence rate, prostate cancer, QOL

## Abstract

**Objectives:**

This study was conducted to evaluate the effect of low-dose chlormadinone acetate, an antiandrogen agent, on the persistence rate of active surveillance in patients with low-risk prostate cancer.

**Methods:**

The study was a multicenter, placebo-controlled, double-blind, randomized controlled trial conducted at 38 sites in Japan. Low-risk prostate cancer patients were randomly assigned to the chlormadinone group or the placebo group and the persistence rate of active surveillance was evaluated for 3 years.

**Results:**

Seventy-one patients in the chlormadinone group and 72 patients in the placebo group were analyzed. The persistence rate of active surveillance [95% CI] at 3 years was 75.5% [62.5–84.6] in the chlormadinone group and 50.1% [36.7–62.2] in the placebo group, showing a significant difference between the groups (*P* = 0.0039). The hazard ratio [95% CI] of the chlormadinone group to the placebo group for discontinuation of active surveillance was 0.417 [0.226–0.770]. The chlormadinone group showed a significant decrease in prostate specific antigen level, testosterone level and prostate volume. The number of positive cores at 12 and 36 months biopsy was significantly lower in the chlormadinone group. The incidence of adverse events was 43.7% in the chlormadinone group and 12.5% in the placebo group. The most common adverse event in the chlormadinone group was constipation in 22.5%, followed by hepatobiliary disorders in 9.9%.

**Conclusions:**

In patients with low-risk prostate cancer, low-dose chlormadinone showed a reduced number of positive cores and prostate volume, and an increased persistence rate of active surveillance (UMIN000012284).

## Teaser

Data from patients with low-risk prostate cancer, chlormadinone, an antiandrogenic agent showed a reduced number of positive cores and prostate volume, and an increased persistence rate of active surveillance.

## Introduction

Prostate cancer (PC) is the second most common cancer next to lung cancer in men, with 1.27 million new cases worldwide and 359 000 deaths in 2018 (1,2). Despite the high incidence, most of patients with early stage PC show slow progression and good prognosis, and the 5-year survival rates for PC were 98% in the USA, 83% in Europe and 99% in Japan (3–5). In addition, the frequency of prostate latent cancer was 19.9% in Asians, 26.7% in Caucasians and 26.2% in Blacks (6). These cancers develop slowly, often over decades, and therefore, the patients may die of other causes without PC being found. Treatments for PC include surgery, radiation therapy, focal therapy, pharmacotherapy (hormonal therapy, chemotherapy, etc.) and active surveillance (AS) (7). Radical prostatectomy (RP) has been reported to have better overall and cancer-specific survival compared with watchful waiting and hormone therapy in randomized controlled trials (8,9). However, RP is an invasive treatment and can have sequelae that affect quality of life (QOL) such as post-operative complications, urinary incontinence and erectile dysfunction. Venderbos *et al.* (10) reported that patients with low-risk PC who had undergone AS had significantly better QOL for urinary function, urinary incontinence and sexual function compared with those who had undergone RP. Furthermore, AS also plays an important role in avoiding overtreatment of PC (11,12). In the USA, the proportion of treatment for localized PC was 47.9% for RP and 8.1% for AS in 2010, but in 2015, RP had decreased to 44.0% and AS had increased to 15.8% (13). According to data from Nara, Japan, the proportion of treatment for low-risk PC was 42.4% for RP and 7.1% for AS in 2004–06, and in 2010–13, RP had decreased to 29.0% and AS had increased to 18.2% (14).

In the long-term follow-up of AS in low-risk PC patients, the rate of discontinuing AS due to cancer progression and switching to other treatments was reported to be about 50% at 10 years (15). Persistence of AS is beneficial for patient QOL; however, the effect of pharmacotherapy on persistence of AS has not been fully investigated.

Chlormadinone acetate (chlormadinone) is an oral antiandrogen agent launched in Japan in 1981 and is indicated for benign prostatic hyperplasia and PC. Chlormadinone has been reported to have anti-cancer effects (16); in untreated PC patients, a complete response was found in 45.2% and a partial response was found in 33.3% according to clinical efficacy criteria reported by Shida *et al.* (17,18). Regarding benign prostatic hyperplasia, chlormadinone has been reported to reduce prostate specific antigen (PSA) level, testosterone level and prostate volume, as well as improved QOL for urinary function (19,20). These effects of chlormadinone may slow the progression of PC and improve the persistence rate of AS. Against this background, we conducted this study to investigate the effect of low-dose chlormadinone on the persistence rate of AS and its safety during long-term administration in patients with low-risk PC.

## Patients and methods

### Design

This study was a multicenter, placebo-controlled, double-blind, randomized controlled trial conducted at 38 sites in Japan from November 2013 to December 2019. This study was registered in University Hospital Medical Information Network—Clinical Trial Registry (UMIN-CTR) (UMIN 0000012284). Patients who met the eligibility criteria and did not meet the exclusion criteria were enrolled and randomly assigned by the enrollment center to the chlormadinone group or the placebo group. The assigned treatment group was concealed until the data were fixed for analysis. The target number of cases in each of the chlormadinone and placebo groups was 110, and the observation period was set to be 3 years. Randomization was performed using a minimization method with age, prostate volume, I-PSS, α1-blocker/anticholinergic agent use and facility as allocation factors. Among the enrolled patients, those who received treatment indicated in the protocol were defined as the full analysis set and were included in the analysis.

This study was conducted in accordance with the ethical principles of the Declaration of Helsinki (revised October 2013) and the Ethical Guidelines for Medical and Health Research Involving Human Subjects established by the Ministry of Health, Labor, and Welfare in Japan. The investigator provided sufficient explanation to the patients before obtaining written informed consent. The study protocol was approved before enrollment in the ethics committee in all study sites.

### Intervention

In Japan, chlormadinone is indicated for PC and benign prostatic hyperplasia, and the approved doses are 100 mg/day for PC and 50 mg/day for benign prostatic hyperplasia. In this study, a low dose of 50 mg (2 × 25-mg tablets) of chlormadinone, corresponding to the dose for benign prostatic hyperplasia, was used. Patients in each group were administered chlormadinone or placebo twice daily for 3 years. During the study period, treatments for PC (surgical therapy, radiation therapy, hormone therapy or chemotherapy) other than those indicated in the protocol of this study were prohibited. Treatment with another antiandrogen agent or a 5α-reductase inhibitor (5αRI) or any invasive treatment for benign prostatic hyperplasia was also prohibited. Treatment with α1-blockers and anticholinergic agents for benign prostatic hyperplasia or difficulty in urination was allowed if necessary.

### Endpoints

The primary endpoint for efficacy was the AS persistence rate. Criteria for discontinuation of AS were defined as any of the following. (i) Disease progression such as a Gleason score ≥ 7, stage ≥T2a, metastasis or increased tumor volume. If there was a clear deterioration in PSA levels or tumor markers (PSA doubling time or PAS density), disease progression was assessed by prostate biopsy, digital rectal examination or ultrasound/imaging. (ii) Onset of difficulty in urination or urinary symptoms requiring invasive treatment. (iii) Cases for which the investigator determines that second-line treatment for PC or benign prostatic hyperplasia is needed.

**Figure 1:**
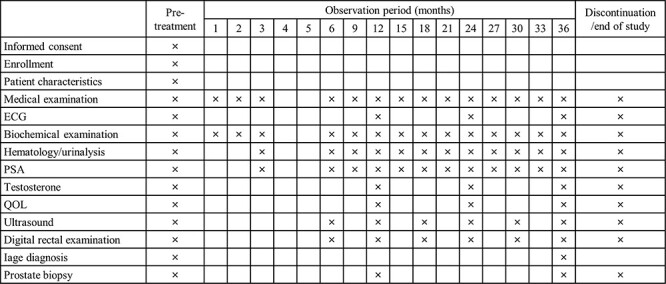
Study schedule, ECG: electro-cardiogram.

The secondary endpoints for efficacy were changes in PSA level, total testosterone level and Gleason score as an indicator of tumor progression. In addition, the rate of no cancer detected, prostate volume and the numbers of biopsy and positive cores were evaluated. Prostate volume was measured using transrectal or transabdominal ultrasound. For prostate biopsy, at least 6 sites of tissue were obtained by transrectal ultrasound-guided systematic biopsy. In this study, pathological diagnosis were performed at each institution according to the standardized criteria. The study schedule is shown in Fig. 1. QOL was evaluated using a domain summary score and domain-specific subscale of the Expanded Prostate Cancer Index Composite (EPIC). For the safety evaluation, adverse events (AEs) were collected in accordance with Common Terminology Criteria for Adverse Events ver. 4.0.

**Figure 2:**
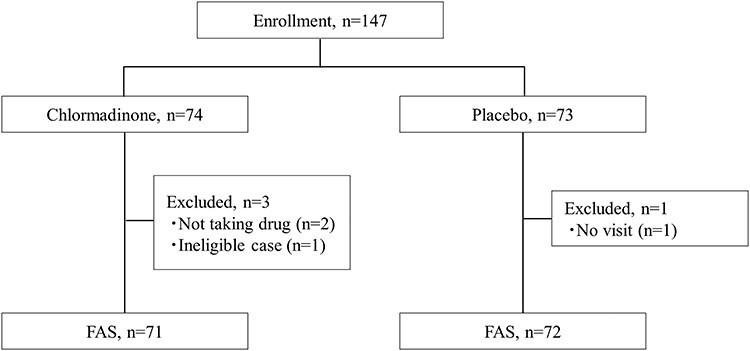
Patient flow.

**Table 1:** Patient characteristics

	Chlormadinone (*n* = 71)	Placebo (*n* = 72)	*P*
Age(years old), median [IQR]	70.0 [67.0, 75.0]	71.0 [67.0, 74.5]	0.6692
Body weight (kg), mean ± SD	64.9 ± 9.3	64.4 ± 9.5	0.747
ECOG PS, *n* (%)			
0	68 (95.8%)	66 (91.7%)	0.4936
1	3 (4.2%)	6 (8.8%)	
TNM classification			
T1c	71 (100.0%)	72 (100.0%)	-
N0	71 (100.0%)	72 (100.0%)	-
M0	71 (100.0%)	72 (100.0%)	-
Gleason score, *n* (%)			
5	5 (7.0%)	3 (4.2%)	0.4936
6	66 (93.0%)	69 (95.8%)	
Number of biopsy core, median [IQR]	12 [10, 12]	12 [10, 16]	0.1291
Prostate volume, *n* (%)			
<30 mL	31 (43.7%)	32 (44.4%)	1.000
≥30 mL	40 (56.3%)	40 (55.6%)	
Complication, *n* (%)			
No	17 (23.9%)	16 (22.2%)	0.8447
Yes	54 (76.1%)	56 (77.8%)	
Concomitant drug, *n* (%)			
No	19 (26.8%)	12 (16.7%)	0.16
Yes	52 (73.2%)	60 (83.3%)	
History of prostate treatment, *n* (%)			
No	65 (91.5%)	66 (91.7%)	1.000
Yes	6 (8.5%)	6 (8.3%)	

ECOG PS, eastern cooperative oncology group performance status; IQR, interquartile range; SD, standard deviation.

**Figure 3:**
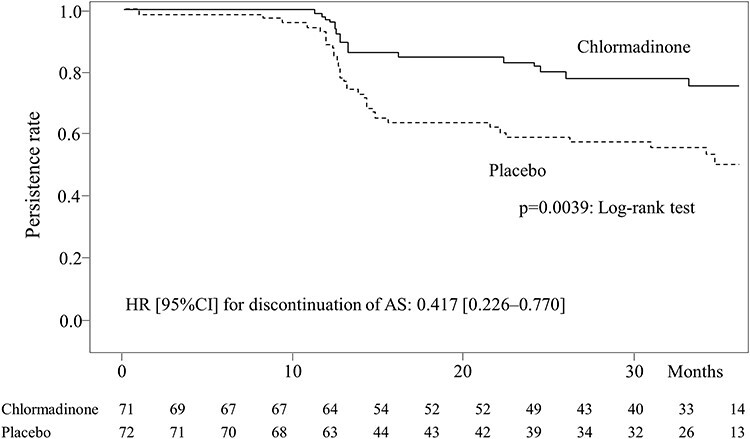
Persistence rates for AS.

### Study subjects

This study enrolled patients who met the eligibility criteria and did not meet any of the exclusion criteria. The eligibility criteria for patients were as follows: (i) diagnosed with PC by histological examination (biopsy), (ii) T1c, N0, M0, Gleason score ≤ 6 and PSA ≤10 ng/mL, (iii) having untreated PC, (iv) within 6 months of starting AS, if already undergone AS, (v) age ≥ 65 years old at enrollment, (vi) ECOG PS 0 or 1 and (vii) submission of written informed consent. The exclusion criteria for patients were as follows: (i) active double cancer, (ii) serious hepatic disorder, serious hepatic disease or serious disease (e.g. chronic renal failure, heart failure, myocardial infarction within 3 months of onset), (iii) serious drug allergy/hypersensitivity, (iv) active urethral genital infection, (v) pain due to prostatitis, (vi) severe lower urinary tract symptoms (LUTS) requiring surgical treatment, (vii) history of prostate surgery, (viii) unable to discontinue treatment with antiandrogen agent or 5αRIs, (ix) not expected to survive for more than 5 years and (x) judged to be unsuitable by investigator.

### Statistics

In a previous report of AS in low-risk PC patients, disease progression was found in 48% of patients in the placebo group at 3 years (21), and thus, the persistence rate of AS in the placebo group after 3 years was assumed to be 50%. Chlormadinone has been shown to suppress the progression of PC for an average of 35.6 months (16) and has been reported to be effective in 88.9% of patients with PC (17). Based on these results, the persistence rate of AS after 3 years in the chlormadinone group was assumed to be 70%. The number of cases required under the conditions of α 0.05 (type 1 error) and power 0.80 was estimated to be 93 cases for each group, and therefore, the number of cases in each group was set to be 110 cases in consideration of unevaluable cases.

The AS persistence rate was estimated using the Kaplan–Meier method, and the log-rank test was used for comparison between groups. The *t*-test was used to compare PSA levels, testosterone levels, prostate volumes and EPIC scores between groups. The Wilcoxon test was used to evaluate the distribution of Gleason score between groups. The significance level was set to be <0.05 on both sides.

## Results

### Patient characteristics

One hundred forty seven patients with PC were enrolled and 74 were assigned to the chlormadinone group and 73 to the placebo group (Fig. 2). Three cases in the chlormadinone group (not taking drug, *n* = 2; ineligible case, *n* = 1) and 1 case in the placebo group (no visit, *n* = 1) were excluded. Finally, 71 cases in the chlormadinone group and 72 cases in the placebo group were analyzed. There were no differences in patient characteristics including age, TNM classification, Gleason score and prostate volume between the two groups (Table 1).

### Endpoints

#### Primary endpoint

In the chlormadinone group, AS was discontinued in 15 of 71 (21.1%) patients due to PC progression. In the placebo group, AS was discontinued in 35 of 72 (48.6%) patients because of PC progression in 32 patients and others in 3 patients. The AS persistence rate [95% CI] was 83.0% [71.4–90.2] in the chlormadinone group and 58.9% [46.3–69.6] in the placebo group at 2 years, and 75.5% [62.5–84.6] in the chlormadinone group and 50.1% [36.7–62.2] in the placebo group at 3 years, showing a significant difference between the two groups (*P* = 0.0039) (Fig. 3). The hazard ratio [HR; 95%CI] for the chlormadinone group to the placebo group for discontinuation of AS was 0.417 [0.226–0.770].

#### Secondary endpoints

**Figure 4:**
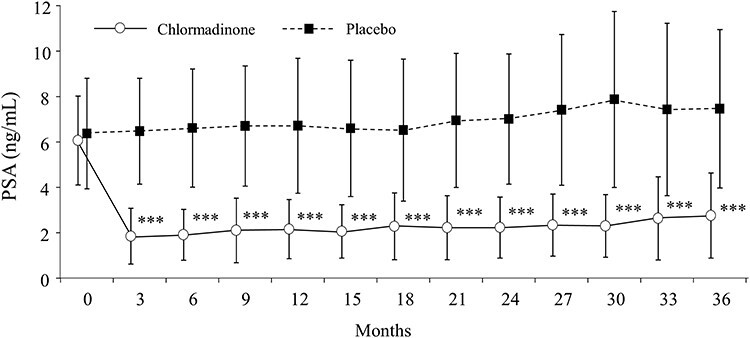
Changes in PSA level, ^*^^*^^*^: *P* < 0.0001.

PSA levels were significantly lower in the chlormadinone group from 3 to 36 months and the difference between the two groups was ~4.0–5.5 ng/mL throughout the study period (Fig. 4). Testosterone levels and prostate volume were significantly lower in the chlormadinone group at 12, 24 and 36 months compared with the placebo group (Table 2). The composition ratio of the Gleason score as tumor progression was significantly different between the groups at 12 months, but not at 36 months (Table 3). The rate for which no cancer detected was relatively high in the chlormadinone group at both 12 months (60.9 vs. 30.2%) and 36 months (64.7 vs. 17.6%). No regional lymph node metastases or distant metastases were found in either group. There was no significant difference between the two groups in the number of biopsy cores at 12 and 36 months; however, the number of positive cores at 12 and 36 months was significantly lower in the chlormadinone group (*P* = 0.0014 and *P* = 0.0073).

**Table 2:** Changes in testosterone level and prostate volume

	Chlormadinone (*n* = 71)	Placebo (*n* = 72)	*P*
Testosterone level (ng/mL), mean ± SD			
Pre-treatment	5.08 ± 2.00	5.60 ± 1.94	0.1273
12 months	1.98 ± 0.82	5.65 ± 1.53	<0.0001
24 months	1.92 ± 0.82	5.66 ± 1.84	<0.0001
36 months	2.65 ± 1.53	5.42 ± 1.68	<0.0001
Prostate volume (mL), mean ± SD			
Pre-treatment	36.4 ± 17.2	35.3 ± 17.5	0.7079
12 months	26.7 ± 13.3	34.1 ± 16.0	0.0147
24 months	21.9 ± 14.0	35.1 ± 15.7	0.0008
36 months	23.7 ± 10.9	39.0 ± 20.7	0.0049

**Table 3:** Histological examination

	Chlormadinone (*n* = 71)	Placebo (*n* = 72)	*P*
No. of patients biopsied, *n* (%)			
Pre-treatment	71 (100.0%)	72 (100.0%)	-
12 months	46 (64.8%)	43 (59.7%)	
36 months	17 (23.9%)	17 (23.6%)	
Gleason score, score (%)			
Pre-treatment			
No cancer detected	0	0	0.459
5	5 (7.0%)	3 (4.2%)	
6	66 (93.0%)	69 (95.8%)	
7	0	0	
8	0	0	
12 months			
No cancer detected	28 (60.9%)	13 (30.2%)	0.0081
5	1 (2.2%)	2 (4.7%)	
6	14 (30.4%)	24 (55.8%)	
7	3 (6.5%)	3 (7.0%)	
8	0	1 (2.3%)	
36 months			
No cancer detected	11 (64.7%)	3 (17.6%)	0.1196
5	0	0	
6	1 (5.9%)	10 (58.8%)	
7	4 (23.5%)	4 (23.5%)	
8	1 (5.9%)	0	
Number of biopsy cores, mean ± SD			
Pre-treatment	11.9 ± 2.9	13.7 ± 6.8	0.0385
12 months	12.1 ± 2.5	13.2 ± 4.7	0.17
36 months	12.4 ± 2.4	12.7 ± 2.2	0.6577
Number of positive cores, mean ± SD			
Pre-treatment	1.4 ± 0.7	1.5 ± 0.9	0.384
12 months	0.7 ± 1.0	1.5 ± 1.4	0.0014
36 months	0.5 ± 0.7	2.1 ± 2.3	0.0073

#### QOL

The urinary summary EPIC score was significantly higher in the chlormadinone group at 36 months (Table 4). The sexual summary score was significantly lower in the chlormadinone group at 12 months, but there was no significant difference between the groups after 24 months. No differences were found in the other EPIC domain summary scores. Regarding the EPIC urinary subscale, the scores for function, burden, incontinence and irritation/obstruction at 36 months were all significantly higher in the chlormadinone group (Table 5). Regarding sexual subscales, the scores for the chlormadinone group were significantly lower after 12 months. Other EPIC domain-specific subscales did not differ between the two groups.

**Table 4:** QOL: EPIC domain summary scores

	Chlormadinone (*n* = 71)	Placebo (*n* = 72)	*P*
Urinary summary scores, mean ± SD			
Baseline	90.6 ± 7.3	91.1 ± 9.2	0.7225
12 months	89.6 ± 10.1	86.1 ± 10.5	0.1310
24 months	92.7 ± 7.4	88.3 ± 10.7	0.0839
36 months	94.2 ± 7.1	81.5 ± 15.0	0.0027
Bowel summary scores, mean ± SD			
Baseline	93.6 ± 8.2	95.0 ± 6.2	0.2562
12 months	93.8 ± 7.3	95.2 ± 5.5	0.3138
24 months	93.3 ± 6.5	95.2 ± 8.8	0.3832
36 months	96.2 ± 4.9	92.2 ± 8.4	0.1053
Sexual summary scores, mean ± SD			
Baseline	40.3 ± 12.9	39.2 ± 12.4	0.6252
12 months	30.9 ± 10.6	37.3 ± 9.4	0.0054
24 months	31.7 ± 11.1	33.6 ± 11.0	0.5293
36 months	30.3 ± 10.4	36.3 ± 10.4	0.1458
Hormonal summary scores, mean ± SD			
Baseline	93.3 ± 7.2	93.9 ± 6.1	0.6138
12 months	93.3 ± 6.2	93.9 ± 5.7	0.6638
24 months	93.1 ± 7.7	93.5 ± 5.0	0.8085
36 months	93.3 ± 8.9	92.4 ± 7.3	0.7460

**Table 5:** EPIC domain-specific subscales

	Chlormadinone (*n* = 71)	Placebo (*n* = 72)	*P*
Urinary subscales, mean ± SD			
Function			
Baseline	95.8 ± 7.4	95.6 ± 7.9	0.8899
12 months	94.9 ± 10.7	90.4 ± 13.4	0.1017
24 months	95.7 ± 8.0	92.7 ± 10.8	0.2355
36 months	97.1 ± 5.4	86.6 ± 16.7	0.0161
Bother			
Baseline	88.6 ± 9.9	87.9 ± 12.0	0.6946
12 months	85.8 ± 12.5	83.0 ± 11.7	0.2988
24 months	90.5 ± 8.7	85.2 ± 12.4	0.0714
36 months	92.1 ± 9.47	77.8 ± 16.2	0.0030
Incontinence			
Baseline	94.8 ± 11.5	94.2 ± 12.0	0.7908
12 months	92.0 ± 17.2	89.5 ± 14.4	0.4810
24 months	84.0 ± 12.1	89.8 ± 18.1	0.3153
36 months	96.1 ± 7.3	85.3 ± 16.4	0.0159
Irritation/obstruction			
Baseline	91.0 ± 7.6	91.0 ± 9.0	0.9889
12 months	90.1 ± 8.5	86.1 ± 10.8	0.0659
24 months	93.3 ± 5.7	89.6 ± 8.3	0.0614
36 months	94.3 ± 7.7	81.1 ± 16.2	0.0038
Bowel subscales, mean ± SD			
Function			
Baseline	92.3 ± 8.9	93.6 ± 8.1	0.3963
12 months	92.9 ± 8.1	94.1 ± 7.1	0.4734
24 months	89.9 ± 10.1	94.3 ± 9.8	0.1104
36 months	93.7 ± 7.6	89.9 ± 10.8	0.2433
Bother			
Baseline	94.8 ± 9.2	96.4 ± 5.4	0.2189
12 months	94.7 ± 7.2	96.4 ± 5.6	0.2462
24 months	96.8 ± 7.1	96.1 ± 8.4	0.7405
36 months	98.3 ± 3.6	94.4 ± 8.2	0.0750
Sexual subscales, mean ± SD			
Function			
Baseline	21.9 ± 18.1	19.5 ± 16.7	0.4270
12 months	9.2 ± 10.6	17.6 ± 14.3	0.0036
24 months	9.5 ± 11.2	15.6 ± 11.0	0.0479
36 months	6.8 ± 11.4	16.3 ± 15.4	0.0459
Bother			
Baseline	81.7 ± 23.1	83.6 ± 23.2	0.6331
12 months	79.8 ± 29.9	81.7 ± 20.7	0.7487
24 months	81.5 ± 28.6	74.0 ± 30.7	0.3528
36 months	83.1 ± 30.8	81.3 ± 25.2	0.8474
Hormonal subscales, mean ± SD			
Function			
Baseline	90.1 ± 11.2	91.3 ± 8.5	0.4891
12 months	91.0 ± 9.7	92.2 ± 7.6	0.5319
24 months	90.9 ± 11.7	91.4 ± 6.0	0.8313
36 months	89.9 ± 14.3	90.9 ± 9.2	0.8049
Bother			
Baseline	96.1 ± 5.6	96.2 ± 5.3	0.9355
12 months	95.3 ± 5.3	95.3 ± 5.5	0.9672
24 months	94.9 ± 6.4	95.3 ± 5.5	0.8075
36 months	96.1 ± 5.5	93.6 ± 9.4	0.3400

#### Safety

AEs were found in 43.7% (31/71) of the chlormadinone group and 12.5% (9/72) of the placebo group. Table 6 shows the AEs found in 3 or more patients in either group and serious AEs (SAEs). The most common AE in the chlormadinone group was constipation (22.5%, 16/71), followed by hepatobiliary disorders (9.9%, 7/71). SAEs were found in 5.6% (4/71) of the chlormadinone group and in 1.4% (1/72) of the placebo group. SAEs found in the chlormadinone group were malignant neoplasms, hepatobiliary disorders and hematomas.

**Table 6:** Adverse events

	Chlormadinone (*n* = 71)	Placebo (*n* = 72)
Incidence of AEs, *n* (%)	31 (43.7%)	9 (12.5%)
AEs occurred in ≥3 cases		
Aspartate aminotransferase increased	3 (4.2%)	0
Alanine aminotransferase increased	3 (4.2%)	0
Malignant neoplasm	3 (4.2%)	1 (1.4%)
Hepatobiliary disorders	7 (9.9%)	1 (1.4%)
Hypertension	0	12 (16.7%)
Urinary frequency	3 (4.2%)	0
Constipation	16 (22.5%)	1 (1.4%)
Dysgeusia	0	5 (6.9%)
Incidence of SAEs, *n* (%)	4 (5.6%)	1 (1.4%)
SAEs		
Malignant neoplasm	2 (2.8%)	1 (1.4%)
Hepatobiliary disorders	1 (1.4%)	0
Hematoma	1 (1.4%)	0

## Discussion

The overall survival rate with AS at 10 years was reportedly 93% in low-risk PC patients, suggesting that low-risk PC patients should undergo AS as a first-line treatment rather than aggressive curative therapy based on adequate risk assessment and life expectancy (15). In this study, low-dose chlormadinone was shown to prolong the persistence rate of AS (HR for discontinuation of AS [95% CI]: 0.417 [0.226–0.770]). In addition, the number of positive cores at 12 and 36 months was significantly lower in the chlormadinone group. These results suggest that antiandrogen therapy with low-dose chlormadinone may prolong the persistence rate of AS through suppression of PC progression. Since AS maintains the patient’s QOL compared with other treatments, prolongation of AS is considered to contribute to the prognosis of PC patients. In Japan, there are few reports of interventional studies on AS and, to our knowledge, this is the first report showing the effect of chlormadinone on the persistency of AS.

In our study, the rate of patients with no cancer detected at 36 weeks was higher in the chlormadinone group than in the placebo group (64.7 vs. 17.6%), but the rate of patients with a Gleason score of 7–8 did not show a clear differences between the groups (29.4 vs. 23.5%). Similar results have been reported with other drugs. Fleshner *et al.* (21) reported on the REDEEM study in which patients with PC undergoing AS were given dutasteride, a 5αRI, or placebo for 3 years, and the rate of PC progression was 48% in the placebo group and 38% in the 5αRI group (*P* = 0.009). In the REDEEM study, the rate of patients with no cancer detected was relatively higher in the 5αRI group than in the placebo group (36 vs. 23%), but the rates of patients with a Gleason score of 7–8 were nearly the same (14 vs. 16%) (21). In addition, Murtola *et al.* (22) reported that antiandrogen therapy with 5αRI reduced the risk of PC for patients with a Gleason score of 2–6 (HR [95%CI]: 0.59 [0.38, 0.91]) but did not affect the risk of PC for patients with a Gleason score of 7–10 (1.33 [0.77, 2.30]). Furthermore, antiandrogen therapy with 5αRI lowers PSA levels in benign prostatic hyperplasia or low-grade PC but shows less PSA-lowering effects in high-grade PC (23,24). These findings suggest that antiandrogen therapy with 5αRI or chlormadinone can be expected to suppress the progression of low-grade PC but may have no or little effect on high-grade PC. Even though antiandrogen therapy lowers PSA levels, it may possibly identify high-grade PC and, therefore, may prolong AS only for low-grade PC, which is a good indication for AS. However, the effects of antiandrogen therapy on the long-term prognosis need further investigation.

There are some differences between our study and the REDEEM study. The number of positive cores has been reported to affect the progression of PC in AS (15,25) and, in the REDEEM study, the percentage of positive cores at final biopsy was relatively lower in the 5αRI group than in the placebo group (13.9 vs. 19.0%), but this was not a significant difference (21). In our study, the number of positive cores in the chlormadinone group at 36 months was about one-fourth that of the placebo group (chlormadinone 0.5 ± 0.7 vs. placebo 2.1 ± 2.8, *P* < 0.0073), which is a significant difference. In addition, the rate of no cancer detected at the final evaluation was 36% for 5αRI and 23% for placebo in the REDEEM study (21), and 64.7% for chlormadinone and 17.6% for placebo in our study, a rate more than three times higher than the placebo group. One of the causes of these differences may be from differences in the target patients. Our study targeted only patients with a tumor class of T1c, whereas the REDEEM study included T1c-T2a patients (21). This suggests that anti-androgenic interventions during AS may be more effective in lower-risk PC populations. Another possible factor is due to the different properties of the drugs. Chlormadinone reduces both dihydrotestosterone (DHT) and testosterone levels, while 5αRI reduces DHT levels and increases testosterone levels (26). Dutasteride, a 5αRI, has been reported to have no significant reduction in prostate volume or PSA level in patients with small prostate volume (<40 mL) or low PSA (27). In another report, the rate of change in PSA level and prostate volume from chlormadinone has been shown to be unaffected by baseline PSA level and prostate volume (28). In the REDEEM study, the mean prostate volume in the 5αRI group was 43.2 mL, suggesting that it may have been a patient population in which the pharmacological effects of 5αRI were not strongly expressed. Furthermore, a non-clinical study showed that chlormadinone has a stronger prostate-reducing effect than 5αRI, and it has been suggested that the reason for the difference between the two drugs is that chlormadinone lowers both DHT and testosterone levels, while dutasteride lowers only DHT level (29). These results suggest that the lowering-DHT effect from dutasteride may be offset by the increase in testosterone level (26). Although the comparison between chlormadinone and 5αRI has not been fully investigated in clinical practice and needs further investigation, it is possible that the different pharmacological actions of the two drugs may have had an effect on the differences between the REDEEM study and this study.

Regarding QOL, the chlormadinone group had significantly higher scores for function, burden, incontinence and irritation/obstruction than the placebo group at 36 months. LUTS has been shown to affect anxiety in PC patients undergoing AS (30). The improvement of QOL in the chlormadinone group may be related to the improvement of LUTS because of the decrease in prostate volume. Regarding sexual subscales, the function score was significantly lower in the chlormadinone group from 12 to 36 months. This was due to a decrease in testosterone levels caused by antiandrogen therapy and is a known effect of chlormadinone. In this study, the dosage of chlormadinone was set to be 50 mg/day, the dose for benign prostatic hyperplasia. PC is often accompanied by benign prostatic hyperplasia, and LUTS is also common in such patients (31). In our study, chlormadinone administration showed prolongation of AS and improvement of urinary QOL. Considering these results, administration of chlormadinone may be one of the options for improving the prognosis of patients when AS is selected for patients with PC coexisting with benign prostatic hyperplasia.

AEs were found in 43.7% of the chlormadinone group and in 12.5% of the placebo group. The most common AE in the chlormadinone group was constipation (22.5%), followed by hepatobiliary disorders (9.9%). Chlormadinone has been used clinically in Japan since 1981, and the results of the safety evaluation in this study were similar to the previously known safety profile. In this study, administration of chlormadinone showed reduced positive core count, improved urinary QOL and increased AS persistency in low-risk PC patients. When monitoring PSA, it is also necessary to consider the PSA-lowering effect of chlormadinone (32).

This study has the following limitations. This study only included patients aged ≥65 years old with clinical stage T1c, N0, M0 PC. The pathological diagnosis were performed at each institution. The pathological findings for the chlormadinone group were obtained from patients undergoing chlormadinone treatment, and the influence of chlormadinone on the Gleason score is unknown. The observation period was 3 years, and so longer term efficacy and safety are unknown. The results of this study need to be interpreted with these factors in mind.

In conclusion, in low-risk PC patients, low-dose chlormadinone reduced prostate volume, the number of positive cores and prolonged the persistence rate of AS while maintaining QOL. Our results showed that low-dose chlormadinone contributes to prolonged AS while maintaining QOL in patients with low-risk PC. However, the effects of chlormadinone on the long-term prognosis need further investigation.
